# Food Safety Management in Primary Schools for Ethnic Groups in Northern Thailand: A PDCA-Based Evaluation

**DOI:** 10.3390/ijerph22091438

**Published:** 2025-09-16

**Authors:** Vivat Keawdounglek, Warapon Paenkhokuard, Anuttara Hongthong

**Affiliations:** Program of Environmental Health, School of Health Science, Mae Fah Luang University, Chiang Rai 57100, Thailand; 5931807016@lamduan.mfu.ac.th (W.P.); anuttara.hon@mfu.ac.th (A.H.)

**Keywords:** food safety, school, ethnic community

## Abstract

Food safety management in rural ethnic schools remains underdeveloped in Northern Thailand. This study evaluates such systems in primary schools using the Plan Do Check Act (PDCA) framework, which can serve as a scalable and culturally sensitive tool to strengthen food safety systems in multi-ethnic school environments. Multi-criteria decision analysis was employed across nine schools representing the Hmong, Lisu, Lahu, Akha, Kamoo, Haw, Mien, Karen, and Lau communities. Data collection included (1) PDCA-based rubric scoring validated by three experts; (2) in-depth interviews and field observations; (3) food and water contamination testing; and (4) microbiological analysis of chefs’ hands and utensils (detected or non-detected). The results showed that only 45% of the schools involved had third-party food safety monitoring, and 45% lacked systems to gather student feedback. None could independently assess chlorine or food residue. *Escherichia coli* contamination was found on chefs’ hands (44%), utensils (56%), and drinking water (33%). Schools C (Lahu) and F (Haw) had the highest and lowest PDCA scores, respectively. Therefore, schools should (1) train employees, (2) work with a third party responsible for monitoring food safety, and (3) establish raw material and contamination self-assessment processes to improve food safety. Implementing PDCA to improve food safety in neglected schools is essential.

## 1. Introduction

A child’s development and general well-being are greatly influenced by their early years of life. According to studies cited in the literature [1,2,3], children’s health might be significantly impacted by their nutrition. Furthermore, the report of the World Health Organization [4] stated that many children are among the estimated 1.6 million people who become ill every day because of consuming contaminated food. Clean food is a major factor in determining one’s health, so obtaining access to clean and nutritious food is an essential human right. In addition, children who are born in certain locations are distinguished by physical traits; those belonging to a particular ethnic group with specific cultural characteristics, such as language, religion, customs, and history, are known as an “ethnic group” [5] and may be at high risk from foodborne disease. Due to a lack of socio-environmental data, our understanding of their impact on disease risk is limited, especially with regard to food safety and food sanitation [6]. Moreover, not all of an ethnic group’s children may receive appropriate welfare. For instance, 70% of migrant children in Guatemala are particularly vulnerable to poverty due to the long-term health and potential impact of malnutrition and illiteracy [7]. A study by Ghanayem et al. [8] estimates that around 500,000 people who belong to ethnic groups in England and Wales have need of special health care. Hence, children belonging to ethnic minorities should have their health monitored, with a particular focus on their quality of life during their basic education, since this will help them grow up to be healthy adults [9].

In Thailand, there are 60 varieties of ethnic groups, and 47% of them are Thai Phuen (They are a Tai ethnic group who use the Phuan language, which is part of the Kra-Tai language family. They live in many provinces of Thailand and originally lived in Laos). Furthermore, there are 731 households of ethnic groups located in Chiang Rai, Thailand. This is the highest level of an ethnic group in the north of Thailand, which consists of the Akha, who have a top ranking among the population in Chiang Rai of approximately 240 households. Meanwhile, the Lahu and Mien are in the second and third rankings of ethnic groups in Chiang Rai, of which there are 207 and 58 households, respectively. Moreover, it has been stated that Chiang Rai has 30 ethnic groups [10]. However, 432 Thai children have suffered food poisoning when consuming contaminated school meals, which they contracted from a school lunch served in a primary school. These children live in an ethnic group in the north of Thailand [11]. This suggests that most schools in ethnically diverse schools should implement a food safety monitoring procedure to prevent the spread of foodborne disease [12,13].

Managing food safety in these settings requires not only technical interventions but also a robust, context-sensitive operational framework. The Plan–Do–Check–Act (PDCA) cycle, first conceptualized by Deming in 1950 [14] and widely adapted in quality, provides such a framework [15,16,17,18,19]. Therefore, the implementation of PDCA principles for the monitoring of food safety management in primary schools located in the ethnic community should be analyzed to establish the main problems affecting food contamination so that school lunches can be improved in quality to prevent the outbreak of foodborne diseases [20]. In fact, PDCA enables a continuous loop of planning, implementation, evaluation, and corrective action, as can be seen in the following explanation [15,16,17,18,19].

1. Plan (P)—This step involves identifying a problem or area for improvement and creating a complete solution. Targets, indicators, and processes or actions need to be clearly established based on evidence and stakeholder needs.
2. Do (D)—This step implements the plan on a small scale or within a specific context. Resource mobilization, training, communication, and activity execution are required. Accurate records and real-time monitoring are essential.
3. Check (C)—The “Do” phase’s results are analyzed to determine efficiency. Data is compared to baseline or predicted results. This evaluation finds gaps, deviations, and unintended consequences.
4. Act (A)—Based on the Check results, process improvements are performed. If an intervention is conducted, it may be standardized or scaled up; if not, the cycle starts again with a new plan.

Although general school health programs may use organized models, there are few empirical studies on PDCA in the ethnic schools of northern Thailand, which are complicated by cultural practices, architectural limits, and resource constraints [21]. These schools should enhance comprehension of their internal procedures and provide scope for localized continuous improvement by adopting a systematic, iterative model for food safety management using PDCA. Adaptability renders it an appropriate tool for compliance. The present study addresses a gap in the literature and practice by showing how PDCA can be used to monitor and improve food sanitation management in primary schools located in an ethnic community in the north of Thailand. This evaluation will recommend some ways in which food hazards, including physical, chemical, and biological contamination in school lunches, can be reduced [22]. In addition, this will not only prevent food contamination in school lunches in this ethnic area, but it will also assist in achieving sustainable development goals, including the children’s good health and well-being [23].

## 2. Materials and Methods

This study focused on the qualitative case study based on primary schools in the ethnic community of northern Thailand. Moreover, the method of using multi-criteria for the analysis was then applied to evaluate the food safety management in the school lunches of primary schools located in the ethnic community. Hence, the materials and methods of this study are described as follows.

### 2.1. Creation of PDCA Criteria

The first step of this research was that of the PDCA criteria development [23] to acquire data from the intended target group. This was created following an investigation of the literature reviews. There are four criteria that are used in the PDCA analysis in this study, which include the following.

Plan (P)—these criteria identify a problem or improvement area of food safety in the primary schools and offer a solution for the school lunches. Therefore, some of the evidence and the stakeholders’ needs should be used to inform targets, indicators, procedures, or actions. The criteria for the Plan (P) should be used by the person responsible for food safety management [24] and the setting of lunch menus for students’ health [1,25].

Do (D)—implements the food safety plan individually or contextually. Resource organizing, training, communication, and activity execution related to food safety management in primary schools are needed. Moreover, real-time monitoring and precise records are important in this process. After the literature review, the D criteria were the food safety training for school lunches [26,27] and the selection of raw materials for school lunches [28,29].

Check (C)—was a comparison of food safety management in primary schools to establish a baseline. This evaluation found gaps, irregularities, and unexpected effects for food safety management in the school lunches. Therefore, these criteria C were included in the health examinations [30,31], the monitoring process for chemical contamination [32], the monitoring process for residual chlorine in the water supply [33,34], and the evaluation of the feedback on the students’ food [35].

Act (A)—was applied by setting up food safety interventions in the school lunches. However, if there were any problems, a new plan for food safety management was created. These criteria were included in the corrective action after feedback from students [36], and responsibility for any chemical and biological contamination in the school lunches was noted [37]. A corrective action plan was checked by an expert, and feedback was given to the school’s director and the chef to improve the quality of the school lunches.

### 2.2. Creation of PDCA Sub-Criteria

When the PDCA criteria were developed, sub-criteria for evaluating food safety management at primary schools were developed using the multi-criteria analysis concept [38]. Table 1 presents the criteria and sub-criteria for this study.

### 2.3. PDCA Criteria and Sub-Criteria Validation

Following the development of the PDCA criteria and sub-criteria, the validation procedure follows. Using the item objective congruence (IOC) process [49], two food safety management experts from Burapha University and Thammasart University, Lampang Campus, Thailand, along with a public health expert from Mae Fah Luang University, Chiang Rai, Thailand, participated in this process and validated the criteria. Following their consideration of each criterion, these experts assigned a score based on criteria congruence, with a score of (−1) indicating that the criterion was unsuitable for measuring food safety management in ethnic community primary schools. In the meanwhile, this measurement might be appropriate for the (0) reference to this criterion. Finally, (1) indicates that it is appropriate for measuring the management of food safety in ethnic community primary schools [50]. The IOC value was then calculated using the following formula [50]:

IOC= ∑XN

where *X* is the score assigned by each expert and *N* is the number of experts.

The average score from the experts, and this criterion and sub-criteria were accepted in this study if it was equal to or greater than 0.6 [51,52]. For this study, the experts approved criteria based on this analysis, and each criterion’s IOC was in the range from 0.611 to 0.988. Therefore, all the criteria were found to be appropriate for measuring the ethnic community’s food safety management.

### 2.4. Locale, Sampling, and Participants

**Figure 1:**
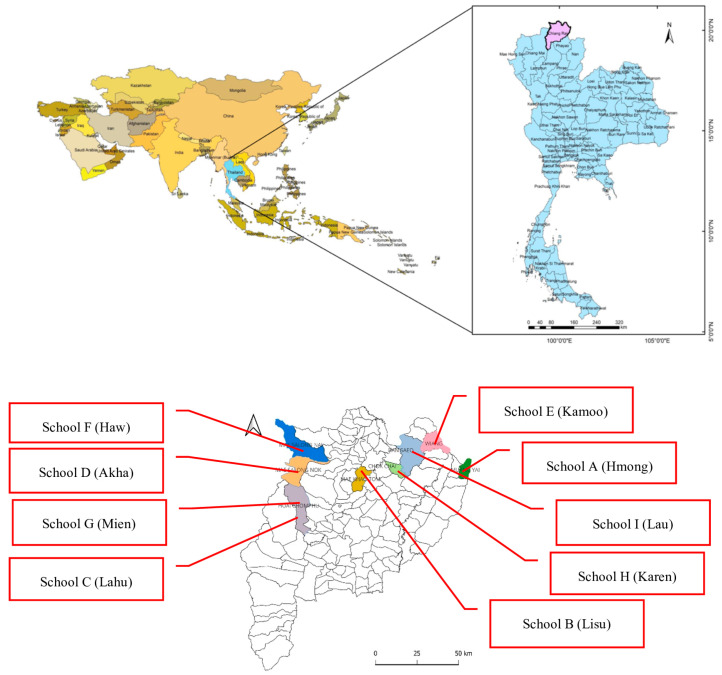
Target group of this study.

After the development of the criteria, the nine ethnic groups that have a high ranking in Chiang Rai were selected because they represent the largest concentration of an ethnic community in northern Thailand, according to the information of the Princess Maha Chakri Sirindhorn Anthropology Centre [53]. Moreover, there were nine ethnic groups, including the Hmong, Lisu, Lahu, Akha, Kamoo, Haw, Mien, Karen, and Lau groups [9]. Therefore, nine primary schools that represent the largest ethnic populations in Chiang Rai were selected as participants in the study [54], as illustrated in Figure 1, following the purposive sampling for the qualitative analysis. For this sampling, following the relevant studies [49,50], we took care to establish similar characteristics to make representative sampling. Therefore, the criteria included the following: (1) They had a chef to make the school lunch, and (2) they had approximately one hundred ethnic students for each primary school. Hence, the target groups of this study were (1) School A, associated with the Hmong group in Wiang Kaen district; (2) School B, linked to the Lisu group in Muang Chiang Rai district; (3) School C, affiliated with the Lahu group in Muang Chiang Rai district; (4) School D, connected to the Akha group in Mae Fah Luang district; (5) School E, representing the Kamoo group in Chiang Khong district; (6) School F, associated with the Haw group in Mae Fah Luang district; (7) School G, linked to the Mien group in Muang Chiang Rai district; (8) School H, affiliated with the Karen group in Doi Luang district; and (9) School I, connected to the Lau group in Chiang Sean district. Data were collected from these participants for the processes shown below.

### 2.5. Data Collection for PDCA Criteria and Sub-Criteria

The researcher used a structural questionnaire to collect the data that corresponded to the PDCA criteria and sub-criteria and interviewed nine directors and nine teachers who provided information about their school lunch programs. Moreover, observation [49,50] of the school lunches was also carried out to confirm the results of these interviews.

### 2.6. The Analysis of Food Contamination of the School Lunches

According to the sub-criteria of 3.2 and 3.3, there were two types of assessments for chemical and biological contamination conducted by the researcher to recheck the effectiveness of food safety management in the primary schools of the different groups. The first was the assessment of the chemical risks associated with the Thailand Health Market standard using test kits from the Department of Medical Science [55] to identify borax, salicylic acid, sodium hydrosulfide, formalin, and pesticide contamination in the three types of raw materials used for school lunches (see Table S1). Secondly, *E. coli* contamination on the hands of the chefs and the utensils was investigated by using SI2 solution, which showed a violet color following a swab and which was then incubated at 37.5 degrees Celsius for 24 h (unit of analysis: detected or non-detected). After that, the positive results from SI2 revealed a yellow color, which was transferred to Eosin Methylene Blue (EMB) agar to confirm *E. coli* contamination. In addition, the most probable number (MPN) technique was then applied to assess the *E. coli* contamination of the drinking water and ice samples in the primary schools [56,57]. Figure S1 provides the details of the analysis of biological contamination in this study.

### 2.7. Final Result

The data from the in-depth interviews, observations, and assessments for chemical and biological contamination in the primary schools were transferred to the scores of the PDCA criteria, and then a total score was calculated. According to Table 1, the total or the maximum score of the PDCA analysis in this study was 17, and the minimum score was zero. Thus, this score was used for the evaluation of food safety management in the primary schools for the different ethnic groups and was divided as follows into three groups following the class interval calculation formula (see in Table S2, Supplementary Materials) [58],

Class interval = [Maximum Score − Minimum Score]/Number in Class

From this calculation, the approximate result of the class interval of 5.7 was obtained. Therefore, the criteria for each class could be identified as follows: (1) A total score of less than 5.7 indicated a poor level of food safety management. (2) A total score from 5.7 to 11.3 indicated a moderate level of food safety management. (3) A total score of more than 11.3 indicated good food safety management [58]. In addition, the inferential statistics, including the independent sample *t*-test at the 0.05 significant level, were then combined.

## 3. Results

### 3.1. The Results

Each primary school in Chiang Rai was in a particular ethnic group, and a person was assigned to be responsible for the management of food safety and lunch service throughout the planning phase. However, a few schools, or 22% (School C in Lahu and School I in Lau), stated that they did not have any food workers from the outsourcing company. To address the school’s lunch and food safety management, they thus assigned a teacher. Additionally, all schools utilized the “Thai School Lunch (TSL)” to schedule lunches and report on the amount and quality of meals that students consumed during their day. Hence, every school in the ethnic group received one point each for having a responsible person for food safety management and for providing a healthy lunch menu.

### 3.2. The Findings of Do

**Figure 2:**
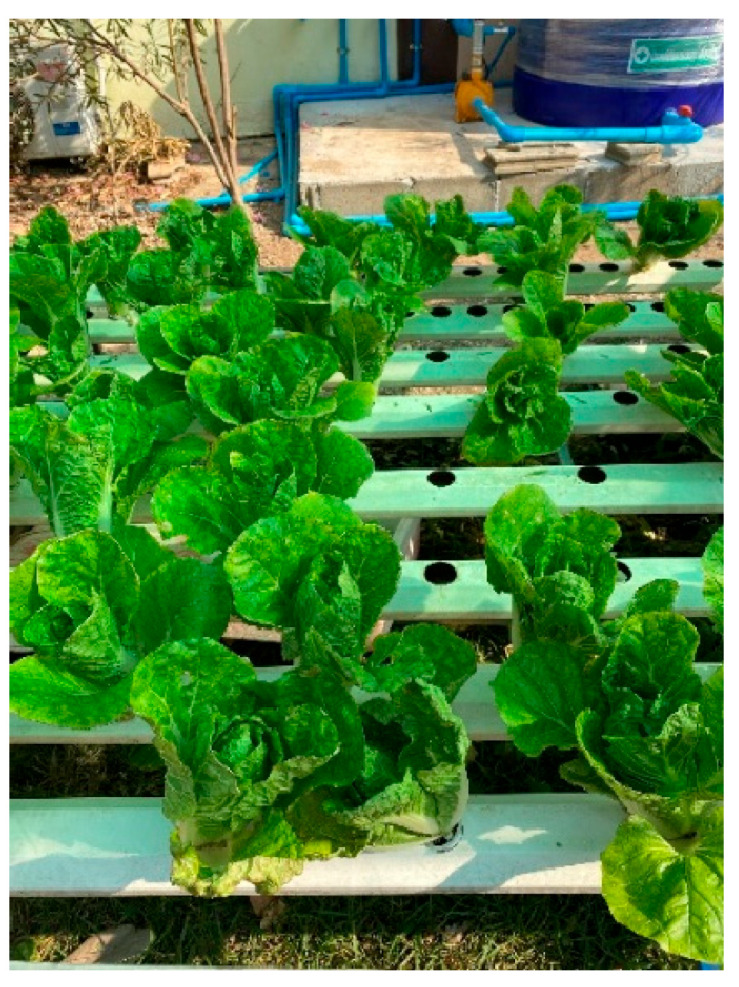
Farming of vegetables in School C.

The food safety training was attended by almost all of the primary schools, or roughly 67% of them; however, schools D and F did not participate because they were unaware of where such training was offered, and they did not know that food handlers were required to attend in order to ensure hygienic and safe food preparation. Regarding raw material farming: Schools C, E, and F, or nearly 34% of the schools, were three primary schools that had a raw material farming technique in place, as can be seen from Figure 2. School C’s rationale for implementing raw material farming was that they received funding from other sponsors to carry it out. Schools E and F stated that their students could practice farming to support their families financially.

### 3.3. The Results of Check

Only five primary schools, comprising schools A, B, C, E, and G, or 56% of the total, participated in the health examination of the food workers. Furthermore, the director of these primary schools declared that to consider renewing the workers’ contracts, it was necessary, in accordance with the terms of the referent (TOR), to verify the food workers’ performance at least once annually. A procedure for monitoring chemical and biological contamination by a third party, such as the district hospital, primary hospital, or local administration, was also in place at just four primary schools: A, C, H, and J. However, none of the primary schools were able to monitor chemical and biological contamination by themselves, as they did not know how this should be performed. Unfortunately, some of the raw materials, including vegetables and fruit for all school lunches, had not been certified by the third party to guarantee food safety. Furthermore, none of the primary schools in the ethnic groups had ever had their residual chlorine levels of water supplies monitored because they may have thought that their school’s water source was safe.

### 3.4. The Results of Act

Following the feedback on the students’ satisfaction with their school lunches at Schools C, D, E, and G, the schools altered their lunch menu the following week in accordance with their needs. By contrast, none of the schools ever modified its lunch menu to accommodate a third party’s results showing chemical or biological contamination because the director and the teacher explained that they had not received those results from a third party.

### 3.5. Chemical and Biological Contamination in the School Lunch

According to the chemical and biological contamination analysis conducted by the researcher, fecal coliform bacteria were present in three primary schools: School E contributed 75 mpn/100 milliliters of fecal coliform bacteria, while schools G and H contributed 3.6 mpn/100 milliliters of fecal coliform bacteria. Additionally, schools E, G, and H had *E. coli* detected in their drinking water. Moreover, five primary schools, approximately 56% of the total, found *E. coli* contamination of their utensils in Schools A, D, F, G, and I. Meanwhile, the chefs’ hands were contaminated with *E. coli* at Schools A, D, E, F, and I (as can be seen in Table S4). Similarly, it was found that an evaluation had been conducted at Schools C, D, E, and G on their students’ food. Students in schools A, D, and E were able to express their satisfaction with their school lunches directly to the teacher responsible, while in School G the students were able to inform the food workers.

### 3.6. PDCA Interpretation

**Figure 3:**
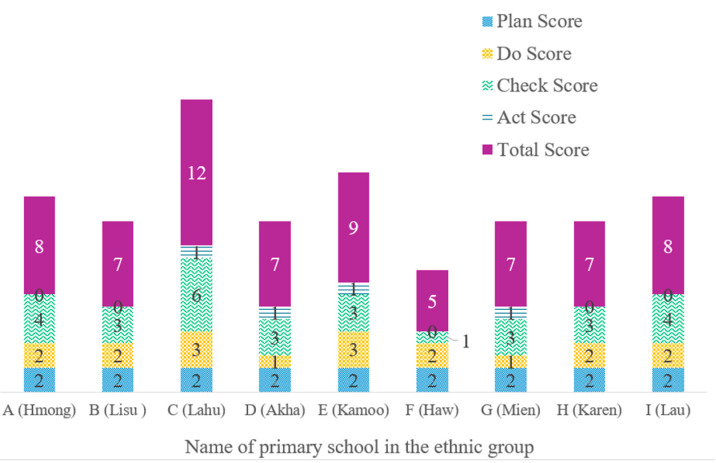
Summary and comparison of Plan, Do, Check, and Act (PDCA) scores for food safety management in primary schools located in the ethnic groups according to the criteria. School C achieved the highest score of 12 in food safety management. School F achieved the lowest score of five in food safety management.

As demonstrated by Table S3 and Figure 3, the results of the PDCA analysis indicated that School C, which served the Lahu, had a good food safety management system in place for its school lunch program. In fact, it received the highest score of 12 out of a total of 17 points. The majority of the primary schools in the ethnic groups, such as School E for the Kamoo, School A for the Hmong, School I for the Lau, School B for the Lisu, School D for the Akha, School G for the Mien, School H for the Karen, and School I for the Lau, had a moderate level of food safety management in place for their lunch programs. These groups had a PDCA score between seven and nine out of a total of seventeen. Conversely, School F for the Haw had the lowest level of food safety management for the lunch program, receiving only five out of a possible seventeen according to the PDCA analysis.

Table S3 and Figure 3 demonstrate that the check during the PDCA cycle had an enormous effect on ethnic primary school food safety management. School C in Lahu received a strong PDCA score in food safety management with a six-point “check” process score. Due to a low enrollment, they received limited funds for school lunch preparation; thus, the teachers prepared it themselves. As shown in the following interview, some of the raw material from school C, including vegetables, had been planted by themselves to minimize costs and avoid using pesticides.

“*Due to the lack of budget to provide a chef in our school, our teachers prepared the school lunch by themselves according to save the cost of school lunch preparation and production. Moreover, we have a vegetable plantation to reduce theses costs*”

As shown in Table S4, School A found *E. coli* on the chef’s hands and utensils despite third-party chemical and biological contamination monitoring. In School E, the chef’s hands and utensils were not monitored by a third party for *E. coli* transmission. It can be assumed that the teachers and chefs were unable to avoid foodborne diseases, and also their personal hygiene during school lunches increased *E. coli* contamination [21,30,31]. According to the independent sample *t*-test, monitoring for chemical and biological contamination and *E. coli* contamination on the chef’s hands were significant factors affecting the ethnic school lunches for the check scores because the *p*-value of the analysis was less than 0.05.

## 4. Discussion

From these findings, it can be assumed that every primary school in the ethnic group is addressing the necessity of teachers or other staff being aware of food safety management when serving lunch. According to the study of Pleerux and Nardkulpat (2023) [59], the personnel involved in food safety management must be held responsible for food quality control and the prevention of the spread of food hazards that could endanger students’ health. Furthermore, children’s overall nutrition should be maintained by using the “Thai School Lunch” program, which should satisfy 30% of the daily recommended dietary allowance (RDA) for nutrients in Thailand and 10% of the RDA for children’s daily milk intake. Healthy lunches must be handled by each primary school by following their teachers’ recommendations for menu planning, food purchasing, preparation, and cooking.

The results show that some ethnic primary schools never received any training in the food safety and sanitation program. As a result, some schools have low scores for food safety management, as in the case of School F. In fact, regulations for food handlers specified in 2018 that food safety training should be conducted in Thailand [10]. To conduct the essential procedures for managing food safety, some schools, namely Schools D, E, and F, need to participate in the food safety training offered by the municipal government or via online learning [27]. Conversely, a few schools, namely Schools C, E, and F, cultivate various foods, including fish, poultry, and vegetables. This enables them to not only produce safe lunch materials [4], but in addition, they can make a profit from selling these materials locally.

As regards the evaluation of the results, a few schools, such as Schools F, H, and I, did not have a health examination of their food workers and handlers. The health examination is a crucial practice, according to a study by Abdisa et al. [3], because food workers’ and handlers’ personal hygiene might have a negative impact if pathogens can contaminate the food. Tables S3 and S4 illustrates how the sub criteria of the chemical and biological contamination monitoring process revealed that most primary schools obtain a score of one since they never performed their own independent monitoring for contamination. Nonetheless, a third party, which included the community hospital and the municipal government, monitored biological and chemical contamination. However, the results of the researcher’s assessment of chemical and biological contamination may be impacted by food safety training and third-party monitoring of biological and chemical contamination. Because School B had a good performance in food safety training, it received a score of two from the researcher’s biological and chemical contamination analysis, while School I was awarded a score of two. School C was awarded a score of two in this analysis because it conducted food safety training, a health examination of the food workers, and enabled a third party to monitor food contamination. Schools C, D, E, and G also participated in this process. According to the study of Orihuela et al. [8], students should be involved in improving the quality of the lunches because they are the biggest stakeholders and reflect how satisfied the students are with their lunches at school. However, none of the primary schools in the ethnic groups monitored the residual chlorine in the water supply. Generally, to disinfect bacteria, such as *E. coli*, the World Health Organization [60] advises that the optimum chlorine residue in a small, provided water supply should be in the range of 0.2 to 0.5 milligrams per liter (mg/L). Furthermore, it was found that the water supply in Schools A, D, F, and G may have used contaminated utensils with *E. coli*, as they should have disinfected their water supply with chlorine before cleaning their utensils.

The last stage of this analysis, was adapted from the study of Ahmad et al. [38], is ACT. According to the feedback from the students, Schools C, D, E, and G improved the quality of their lunch services. Nonetheless, as regards chemical and biological contamination, none of the primary schools improved its management of food safety. As a result, the researcher encouraged the primary schools in the ethnic groups to participate in a meeting about the lessons learned from this study. During this meeting, participants learned about the procedures for monitoring food safety and the methods used for maintaining and enhancing the food safety management process. Additionally, an invitation will be extended to the research team to carry out improvements in food safety management in the upcoming academic year. From the overall PDCA evaluation of the food safety of school lunches in the ethnic community, School C, which had stronger internal feedback and external monitoring, had a better PDCA performance and lower food contamination. Schools lacking such frameworks (e.g., School F) had lower hygiene and adherence. This supports the claim that PDCA improves procedures and establishes school actor responsibility and accountability. In this study, all primary schools that lacked third-party certification for food safety in the school lunch did not receive any information about the need for the assistance of a third party for the certification. Generally, the school’s director or the teacher who is responsible for school lunches can contact the relevant local government, including the local administration, local hospital, or the provincial hospital, to collect the necessary samples for this certification. Therefore, all primary schools should contact or request food safety certification from their local government.

For food safety management worldwide, Sweden and other nations have demonstrated that organized audit-feedback systems, including PDCA, can enhance school lunch quality. Patterson et al. [61] found that Swedish schools using an automated self-monitoring tool improved nutritional compliance over time in a large-scale study. “Cyclical self-evaluation systems raised compliance by 30% with each audit. Through standardized monitoring and assessment by the Ministry of Education, Japan’s school meal program also incorporated PDCA concepts. These techniques assure sanitation, menu quality, and stakeholder feedback, according to Kojima et al. [62]. Both Swedish and Japanese systems are centralized and resource-rich. This study’s schools face diverse challenges: poor infrastructure, ethnic-linguistic variety, and fluctuating local government support. These settings demonstrate PDCA’s versatility, especially when tailored to cultural and logistical needs. Furthermore, the PDCA cycle improved school food safety only when schools systematically included feedback loops, involving parents and nutritionists in the Act phase, and achieved better hygiene outcomes when they conducted community-based food inspections and post-incident reviews as part of the Check and Act processes. In contrast, the Thai schools in this study appear to be operating PDCA in a fragmented form, heavily skewed toward planning but lacking a process of checking to include the detection of biological and chemical contamination, affecting the lowest levels of food safety management in primary schools.

In summary, this study presented three distinct types of recommendations for the future implementation of food safety management of school lunches: (1) those schools with poor scores should attend food safety training to improve personal hygiene for the schools’ lunch preparation and cooking [30]; (2) those schools with moderate scores should monitor food contamination with the help of a third party, and encourage the health examination of food workers and handlers, and create a monitoring process to assess the feedback on school lunches and resolve any issues that arise and; (3) those schools with good scores should monitor food contamination for themselves and implement procedures to maintain their high standards of food safety management [3,31]. In addition, the relevant sectors, including public health volunteers, local government, or the provincial public health office, should cooperate to improve the quantity and quality of food safety management, especially in the primary schools that had the lowest scores for the PDCA evaluation. Despite these encouraging results, PDCA’s use in ethnically diverse educational settings is understudied. The present study addresses that gap and encourages more research. For further study, this PDCA evaluation should include culturally appropriate practices, local food habits, and community engagement to work in rural schools.

In conclusion, three main categories of recommendations are proposed by this study for the future implementation of food safety management for school lunches: (1) attending food safety training to enhance personal hygiene in the school’s lunch preparation and cooking (2) having a third party monitor food contamination; promoting health examinations for food workers and handlers, and establishing a monitoring process for any raw materials used in school lunches; and (3) monitoring food contamination independently and implementing procedures to uphold high standards of food safety management for school lunches. The PDCA-based strategy also emphasizes the need for scalable, culturally adapted interventions to ensure food safety in ethnic schools while respecting local traditions and public health requirements. For long-term sustainability, feedback loops between external assessments and school procedures should be strengthened, and local contamination monitoring and hygiene management skills developed.

## 5. Conclusions

The Ministry of Education’s policy has established school lunches as a vital component of primary education in Thailand. Moreover, school lunches help children in their development towards adolescence. However, Chiang Rai in Thailand has an enormous number of primary schools located in ethnic communities, which are in remote areas that require difficult means of transport. The management of food safety in the context of primary schools is consequently impacted by this situation. The PDCA analysis with rubric scores was therefore developed to evaluate food safety management in the primary schools of ethnic groups in Chiang Rai, Thailand, which included the Hmong, the Lisu, the Lahu, the Akha, the Kamoo, the Haw, the Mien, the Karen, and the Lau.

This study found that every school appointed a staff member or teacher to be responsible for food safety, and they used the Thai School Lunch (TSL) program to ensure the school meals met the nutritional requirements throughout the planning phase. In the phase of doing, while some schools were able to grow some of the ingredients needed to make lunch, only a few schools in this region had a food safety training program in place. As regards checking, even though over 66% of the primary schools in the ethnic groups required health examinations, less than 45% of this target group used a third party to monitor food contamination, and there was a lack of feedback from the students. In addition, none of the schools monitored food contamination and residual chlorine independently. The percentage of *E. coli* contamination on the chefs’ hands, utensils, and the researcher’s analysis of the drinking water was 44%, 56%, and 33%, respectively. In the final phase (Act), it was found that 44% of the primary schools improved the standards of their school lunches after receiving feedback from their students. Unfortunately, because no information or results were provided to the schools following the food contamination analysis, none of the schools has ever been able to improve the quality of their lunches. According to this study, School F in the Haw community had the poorest rating for food safety management, while School C in the Lahu community had an excellent score.

In conclusion, three main categories of recommendations are proposed by this study for the future implementation of food safety management for school lunches: (1) attending food safety training to enhance personal hygiene in the school’s lunch preparation and cooking (2) having a third party monitor food contamination; promoting health examinations for food workers and handlers; and establishing a monitoring process for any raw materials used in school lunches; and (3) monitoring food contamination independently and implementing procedures to uphold high standards of food safety management for school lunches. The PDCA-based strategy also emphasizes the need for scalable, culturally adapted interventions to ensure food safety in ethnic schools while respecting local traditions and public health requirements. For long-term sustainability, feedback loops between external assessments and school procedures should be strengthened, and local contamination monitoring and hygiene management skills developed.

## Figures and Tables

**Table 1:** Criteria and sub-criteria for this study.

Criteria	Sub Criteria	Rubric Score	References
1. Plan			
	1.1 Responsible Person for Food Safety Management	1 = Primary school is assigned a responsible person for food safety management 0 = Primary school is not assigned a responsible person for the food safety management	[24,39,40]
	1.2 The Setting of a Lunch Menu for Students’ Health	1 = A lunch meal designed to assist children’s health is planned for the primary school 0 = A lunch meal is not designed to assist children’s health in the primary school	[1,25,41]
2. Do			
	2.1 Food Safety Training	1 = Food workers in primary schools received training on safe food handling procedures 0 = Food workers in primary schools did not receive training on safe food handling procedures	[26,27,42]
	2.2 Selection of Raw Materials for School Lunch	2 = Raw materials are farmed by primary school students 1 = Raw materials are received from other outsources 0 = The sources of raw materials could not be clearly identified	[28,29,42]
3. Check			
	3.1 Health Examination	1 = Food workers in primary schools have a health examination at least once a year 0 = Food workers in primary school do not have a health examination at least once a year	[30,31]
	3.2 Monitoring Process for Chemical Contamination	2 = Both primary schools and a third party have been mandated to monitor chemical contamination 1 = Primary schools or a third party have been mandated to monitor chemical contamination 0 = There is no procedure in primary schools to check on chemical contamination	[32,43]
	3.3 Monitoring Process for Biological Contamination	2 = Both primary schools and a third party have been mandated to monitor biological contamination 1 = Only a third party has been mandated to monitor biological contamination 0 = There is no procedure in primary schools to check on biological contamination	[26,32,44]
	3.4 Monitoring Process for residual chlorine in water supply	2 = Both primary schools and a third party have been appointed to monitor the residual chlorine in the water supply 1 = A primary school or a third party have been appointed to monitor the residual chlorine in the water supply 0 = No procedure has been implemented in the primary schools to check on residual chlorine	[33,45]
	3.5 Process to Measure the Feedback on Students’ Food (including the satisfaction of students, the follow-up process for foodborne disease after they ate a school lunch)	1 = The primary school evaluates the feedback on their food 0 = The primary school does not evaluate the feedback on their food	[35,46]
Act			
	4.1 Corrective Action after Feedback from students	1 = The primary school creates an action plan responding to the feedback on their food 0 = The primary school does not create an action plan responding to the feedback on their food	[36,47]
	4.2 Responsibility for Chemical and Biological Contamination	1 = They solve this contamination problem when the chemical and biological contamination is identified 0 = They do not solve this contamination problem after the chemical and biological contamination is identified	[37,42,48]

## Data Availability

The dataset is available on request from the authors.

## Supplementary Materials

The following supporting information can be downloaded at: https://www.mdpi.com/article/10.3390/ijerph22091438/s1, Table S1: Type of raw materials sampling for chemical analysis with the test-kits; Table S2: Class interval calculation for criteria interpretation; Figure S1: Sampling for biological hazard assessment; Table S3: A comparison of the PDCA scores for food safety management in the primary schools located in the ethnic groups; Table S4: Independent samples *t*-test for the check score of the ethnic school lunch.

## Abbreviations

The following abbreviations are used in this manuscript:

P	Plan
D	Do
C	Check
A	Act
IOC	Item Objective Congruence
ND	Non-Detection of *E. coli*
DT	Detection of *E. coli*
