# Supplementary material for: Food Safety Management in Primary Schools for Ethnic Groups in Northern Thailand: A PDCA-Based Evaluation
Source: Int J Environ Res Public Health. 2025 Sep 16;22(9):1438. doi: 10.3390/ijerph22091438 (PMC12469511; doi:10.3390/ijerph22091438)
Supplement: Supplementary file 1 [file ijerph-22-01438-s001.zip › ijerph-3798479-supplementary.pdf]

Supplementary Material

Table S1. Type of raw materials sampling for chemical analysis with the test-kits

Type of chemical test-kits	Type of samples (select only 3 types)
Borax	-Ground Pork<br>-Ground Chicken<br>-Meat Ball<br>-Sausage<br>-Ham<br>-Snake
Salicylic Acid	-Curry Paste<br>-Blend Chily<br>-Garlic<br>-Ground Nut
Formalin	-Sea Food<br>-Crispy Pickle Squid<br>-Some of vegetable
Sodium Hydrosulfide	-Bean Sprout<br>-Pickled bamboo shoots<br>-Some of animal entrail<br>-Salt<br>-Sugar
Pesticide (by MJPK test-kits)	-Vegetable<br>-Fruit

Supplementary Information

Figure S1 Sampling for biological hazard assessment

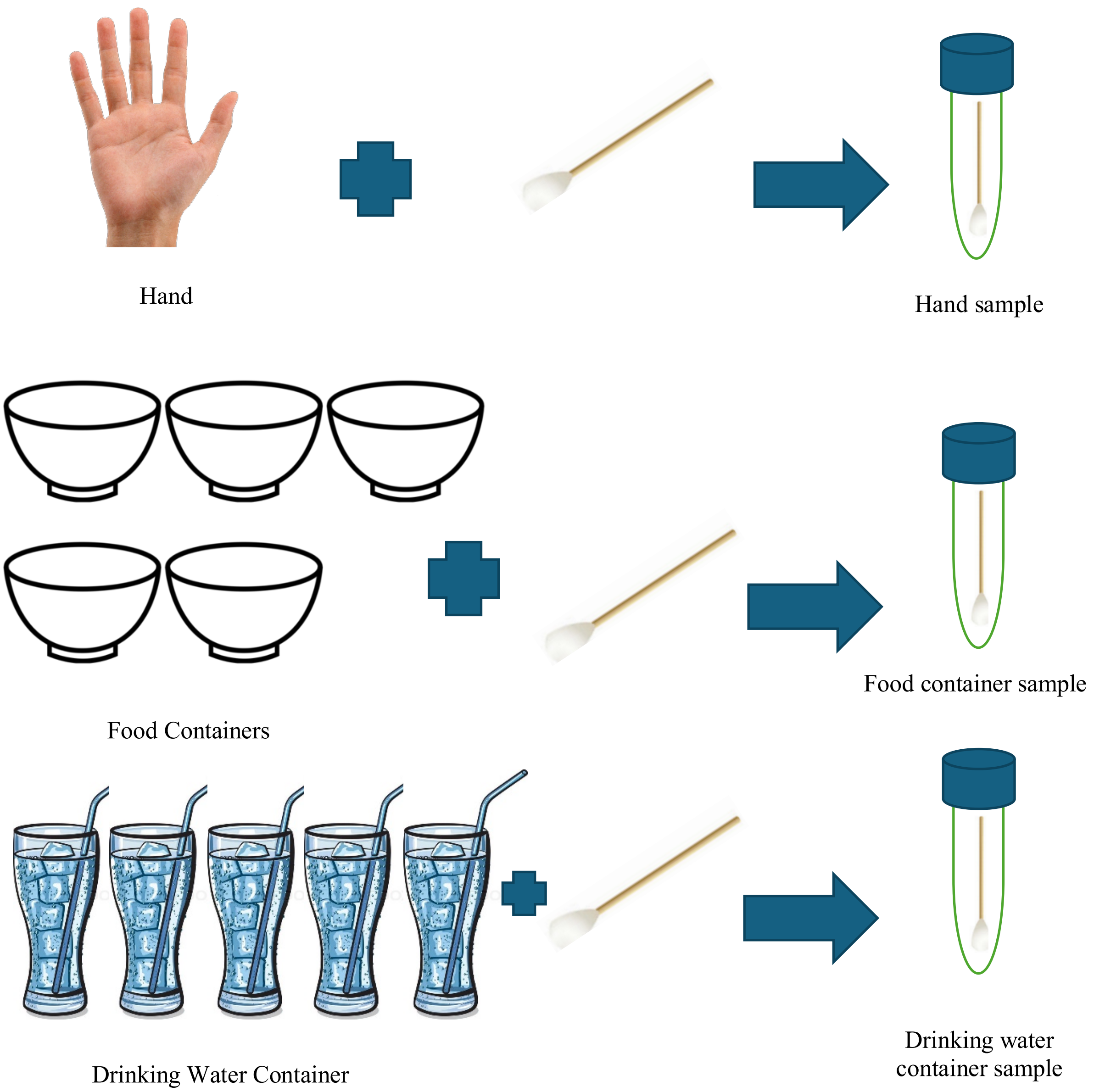

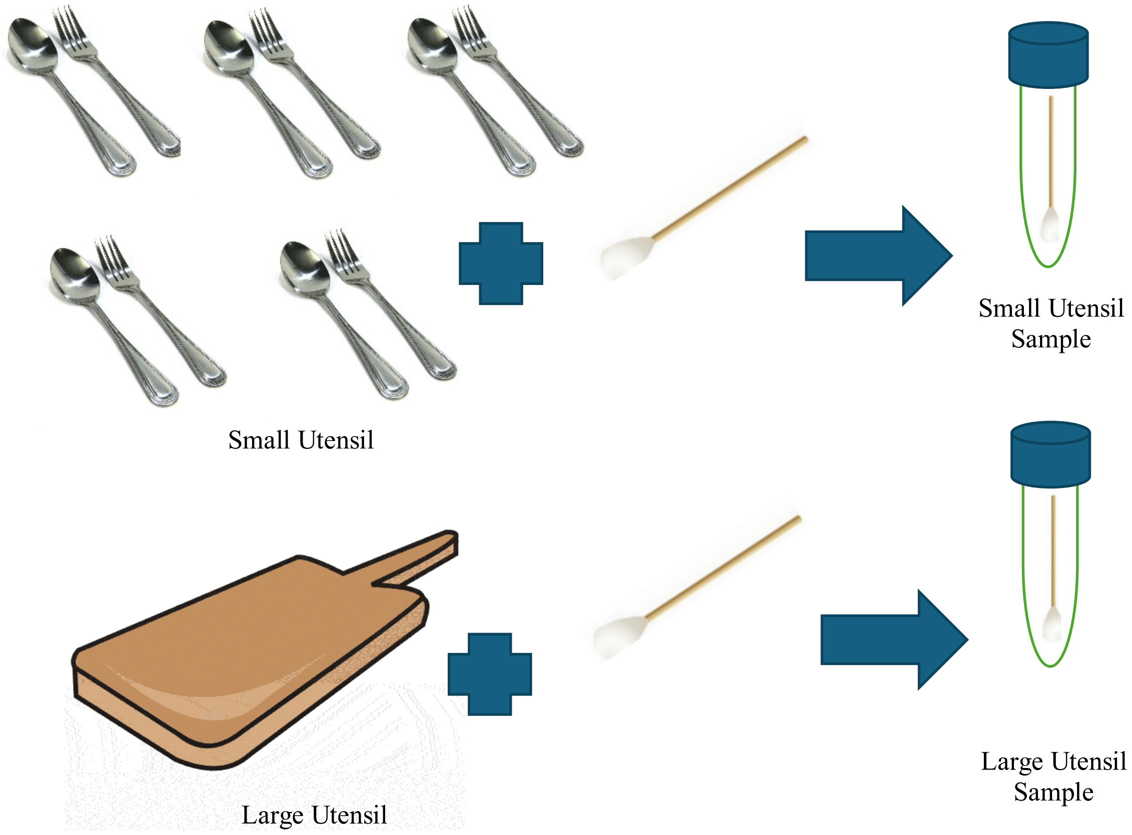
Small Utensil Sample
Small Utensil
Large Utensil Sample
Large Utensil

Supplementary Material

Table S2. Class interval calculation for criteria interpretation

**Item**	**The information for calculation and interpretation**
Formular	[Maximum Score – Minimum Score]/ Number of Class
Detail of this calculation	-Number of class = three classes<br>-Minimum Score from PDCA criteria = zero<br>-Maximum Score from PDCA criteria = 17
Calculation	Class interval<br>= [17+0]/3<br>= 17/3<br>= 5.66666<br>= 5.7 (approximately)
Class order	0.0 -5.69 = Poor level<br>5.70 – 11.40 = Moderate level<br>> 11.40 = Good level

Supplementary Materials

Table S3. A comparison of the PDCA scores for food safety management in the primary schools located in the ethnic groups

Main Criteria	Sub criteria	Rubric Score	Score								
			A	B	C	D	E	F	G	H	I
1.Plan	1.1 Responsible Person for Food Safety Management	1 = Primary school is assigned a responsible person for food safety management<br>0 = Primary school is not assigned a responsible person for food safety management	1	1	1	1	1	1	1	1	1
	1.2 The Setting of a Lunch Menu for Students' Health	1 = A lunch menu designed for children's health is planned for the primary school.<br>0 = A lunch menu is not available for children's health.	1	1	1	1	1	1	1	1	1
2.Do	2.1 Food Safety Training	1= Food workers in primary schools received training on safe food handling procedures<br>0 = Food workers in primary schools did not receive training on safe food handling procedures	1	1	1	0	1	0	0	1	1
	2.2 Selection of Raw Materials for School Lunch	2 =Raw materials are farmed by primary school students.<br>1 = Raw materials are received from other outsources	1	1	2	1	2	2	1	1	1
3.Check	3.1 Health Examination	1= food workers in primary schools have a health examination at least once a year.<br>0= food workers in primary schools do not have a health examination at least once a year.	1	1	1	1	1	0	1	0	0

Main Criteria	Sub criteria	Rubric Score	Score								
			A	B	C	D	E	F	G	H	I
	3.2 Monitoring Process for Chemical Contamination	2 = Both primary schools and a third party have been mandated to monitor chemical contamination.<br>1 = a primary school or a third party have been mandated to monitor the chemical contamination.<br>0 = There is no procedure implemented in primary schools to check on chemical contamination	1	0	1	0	0	0	0	1	1
	3.3 Monitoring Process for Biological Contamination	2 = Both primary schools and a third party have been mandated to monitor biological contamination.<br>1 = Only a third party has been mandated to monitor biological contamination.<br>0 = No procedure has been implemented in primary schools to check on biological contamination	1	0	1	0	0	0	0	1	1
	3.4 Monitoring Process for residual chlorine in water supply	2 = Both primary schools and a third party have been appointed to monitor the residual chlorine in the water supply.<br>1 = a primary school or a third party have been appointed to monitor the residual chlorine in the water supply.<br>0 = No procedure has been implemented in the primary schools to check on residual chlorine	0	0	0	0	0	0	0	0	0
	3.5 Process to Measure the Feedback on Students' Food (including the satisfaction of students, the follow-up process for	1 = Primary schools evaluate the feedback on their food<br>0 = Primary schools do not evaluate the feedback on their food	0	0	1	1	1	0	1	0	0

Main Criteria	Sub criteria	Rubric Score	Score								
			A	B	C	D	E	F	G	H	I
	foodborne disease after they ate a school lunch)										
	Monitoring process for chemical and biological contamination from the researcher	2 = no biological or chemical contamination<br>1 = detection of biological or chemical contamination	1	2	2	1	1	1	1	1	2
**4. Act**	4.1 Corrective Action After Feedback from Students	1 = The primary school create an action plan responding the feedback on their food<br><br>0 = The primary school does not create an action plan responding the feedback on their food	0	0	1	1	1	0	1	0	0
	4.2 Responsibility for Chemical and Biological Contamination	1 = They solve this contamination after the chemical and biological contamination are monitored<br>0 = They do not solve this contamination after the chemical and biological contamination are monitored	0	0	0	0	0	0	0	0	0
**Total Score**		**17**<br>*0 – 5.69 = Poor Management (P)*<br>*5.70 – 11.39 = Moderate Management (M)*<br>*11.40 – 17.00 = Good Management (G)*	**8 (M)**	**7 (M)**	**12 (G)**	**7 (M)**	**9 (M)**	**5 (P)**	**7 (M)**	**7 (M)**	**8 (M)**

Supplementary Material

Table S4 Independent samples t-test for the check score of the ethnic school lunch

Ethnic primary School	Score of check	Criteria of Check					*E.coli* contamination	
		Health Examination	monitoring for chemical contamination from third party	monitoring for biological contamination from third party	Monitoring of water supply	Student feedback evaluation	Chief's hands	Utensils
A (Hmong)	4	Yes	Yes	Yes	No	No	DT.	DT.
B (Lisu)	3	Yes	No	No	No	No	ND.	ND.
C (Lahu)	6	Yes	Yes	Yes	No	Yes	ND.	ND.
D (Akha)	3	Yes	No	No	No	Yes	DT.	DT.
E (Kamoo)	3	No	No	No	No	Yes	ND.	ND.
F (Haw)	0	Yes	No	No	No	No	DT.	DT.
G (Mien)	3	Yes	No	No	No	Yes	DT.	DT.
H (Karen)	3	No	Yes	Yes	No	No	ND.	ND.
I (Lau)	4	No	Yes	Yes	No	No	ND.	DT.
p-Value		0.197	0.036*	0.036*	-	0.399	0.010*	0.062

Notice: ND. is non-detected, DT. is detected
